# Visualization of skin microvascular dysfunction of type 1 diabetic mice using *in vivo* skin optical clearing method

**DOI:** 10.1117/1.JBO.24.3.031003

**Published:** 2018-08-17

**Authors:** Wei Feng, Rui Shi, Chao Zhang, Shaojun Liu, Tingting Yu, Dan Zhu

**Affiliations:** a Huazhong University of Science and Technology, Wuhan National Laboratory for Optoelectronics, Britton Chance Center for Biomedical Photonics, Wuhan, Hubei, China; b Huazhong University of Science and Technology, School of Engineering Sciences, Collaborative Innovation Center for Biomedical Engineering, MoE Key Laboratory for Biomedical Photonics, Wuhan, Hubei, China

**Keywords:** diabetes, skin optical clearing, microcirculation, vascular dysfunction, hyperspectral imaging, laser speckle imaging

## Abstract

To realize visualization of the skin microvascular dysfunction of type 1 diabetic mice, we combined laser speckle contrast imaging and hyperspectral imaging to simultaneously monitor the noradrenaline (NE)-induced responses of vascular blood flow and blood oxygen with the development of diabetes through optical clearing skin window. The main results showed that venous and arterious blood flow decreased without recovery after injection of NE; furthermore, the decrease of arterious blood oxygen induced by NE greatly weakened, especially for 2- and 4-week diabetic mice. This change in vasoconstricting effect of NE was related to the expression of α1-adrenergic receptor. This study demonstrated that skin microvascular function was a potential research biomarker for early warning in the occurrence and development of diabetes. The *in vivo* skin optical clearing method provides a feasible solution to realize visualization of cutaneous microvessels for monitoring microvascular reactivity under pathological conditions. In addition, visual monitoring of skin microvascular function response has guiding significance for early diagnosis of diabetes and clinical research.

## 1 Introduction

Diabetes mellitus is a chronic, systemic disease characterized by hyperglycemia. Abnormal insulin secretion or disordered insulin biofunction can lead to high blood glucose, which will cause vascular dysfunction and immune disorders,^1^^,^^2^ and even severe complications to various organs, including brain, eyes, and skin.^3^^–^^5^ The abundant vascular network in skin provides an accessible and common vascular bed for assessing vascular hemodynamic response.^6^^,^^7^ In recent years, the skin vascular function started to draw widespread attention as a potential research biomarker to predict cardiovascular diseases^8^ and diabetic retinopathy^9^ or as a prognostic marker for evaluating drugs effect on the microcirculation.^10^ Previous studies showed diabetes could cause various skin vascular changes, including microvascular structure,^11^ vascular density and diameter,^12^ blood flow and blood oxygen,^13^^,^^14^ and vascular permeability.^12^ However, it is still unclear how the skin microvascular dysfunction will change with the development of diabetes. Due to the fact that diabetes is a chronic metabolic disease, it is necessary to *in vivo* monitor the skin microvascular function along with development of diabetes. It will be helpful for understanding the influence of progressive diabetes on skin vascular dysfunction and the interventional therapy strategies of diabetes.

Researchers employed various reactivity tests to assess the skin vascular functional response under diabetic condition,^10^^,^^15^^,^^16^ in which the pharmacological interventions such as acetylcholine, sodium nitroprusside, and noradrenaline (NE) were applied.^8^^,^^10^ Some noninvasive *in vivo* optical imaging techniques have been proposed to monitor microvascular reactivity tests,^17^ including laser Doppler technique,^18^ photoacoustic imaging,^13^ laser speckle contrast imaging (LSCI),^19^ hyperspectral imaging (HSI), etc.^20^^,^^21^ Low-cost LSCI and HSI have good temporal resolution, which allow us to noninvasively monitor wide-field peripheral microcirculatory blood flow and blood oxygen in real time. There may be some differences in vascular responsiveness to reactivity tests between arteries and veins with the development of diabetes. Thus, the imaging method with high spatial–temporal resolution is required for delivering an objective evaluation of microvascular reactivity tests.^22^ But there is a technical challenge to study targeted skin vasculature directly.^8^ High scattering of skin is a barrier that greatly limits optical imaging performance, which leads to poor visualization of skin microvascular reactivity. This poor visualization of skin microvascular functional response might lead to misunderstanding of complex mechanisms underlying the regulation of reactivity tests. Therefore, some skin imaging windows were established with surgery for improving the performance of imaging cutaneous blood vessels or cells.^23^^,^^24^ Actually, the surgery will inevitably lead to proinflammatory stimulus^25^ and even affect the vascular structure and function. Thus, we need a suitable and noninvasive skin imaging window for visualization of skin vascular dysfunction. Fortunately, *in vivo* skin optical clearing method has been developed and implemented in recent years,^26^^–^^28^ which has excellently enhanced the imaging performances on various optical imaging modalities, including optical coherence tomography (OCT),^29^^–^^31^ photoacoustic microscopy,^32^^,^^33^ LSCI,^34^^–^^39^ HSI,^40^ and confocal microscopy.^41^^–^^43^

In this study, based on the alloxan-induced type 1 diabetic (T1D) mice model, we evaluated NE-induced skin vascular responses (including blood flow and blood oxygen) along with the development of diabetes by LSCI and HSI, with the help of *in vivo* skin optical clearing method we developed previously.^41^

## 2 Materials and Methods

### 2.1 Type 1 Diabetes Mellitus Animal Model

The Balb/c mice have been widely used in animal physiopathological experiments. Here, according to the previous reported diabetic mice models,^44^^–^^46^ adult male Balb/c mice were employed. Mice were intraperitoneally administered with 150-mg/kg alloxan (30 mg/mL), respectively, for four days after 4 h of fasting each day. The blood glucose was detected to validate the T1D mice model depending on whether the fasting blood glucose was higher than 7 mmol/L. Different groups of T1D mice at each diabetic stage were classed depending on diabetic duration and named 1-, 2-, and 4-week T1D mice. All experimental procedures were performed according to animal experiment guidelines of the Experimental Animal Management Ordinance of Hubei Province, China, and the guidelines from the Huazhong University of Science and Technology (HUST), which have been approved by the Institutional Animal Ethics Committee of HUST.

### 2.2 In Vivo Skin Optical Clearing Method

Here, we used the *in vivo* dorsal skin optical clearing method^41^ to enhance the imaging qualities of LSCI and HSI. The dorsal skin optical clearing agent (DSOCA) was a mixture of PEG, thiazone, and sucrose [67.1% (wt./wt.)]. The mice were anesthetized with the mixture of 2% α-chloralose and 10% urethane (8 mL/kg) via intraperitoneal injection. Then, the dorsal hair was shaved with electric hair clipper and the residual hair was removed thoroughly with local application of depilatory cream, this method of hair removal referenced to the reported work.^41^ During the experiment, the mouse was placed on a heating pad for maintaining body temperature at 36.5°C to 37.5°C. To establish the optical clearing skin window, DSOCA was topically applied on the dorsal region of interest for 15 min; the treatment time of DSOCA was the same in all experiments. To avoid the effect of many experimental factors, each mouse was used for only one experiment and all experimental conditions were kept as consistent as possible.

### 2.3 Laser Speckle Contrast Imaging and Hyperspectral Imaging for Imaging Vascular Blood Flow and Blood Oxygen

**Fig. 1:**
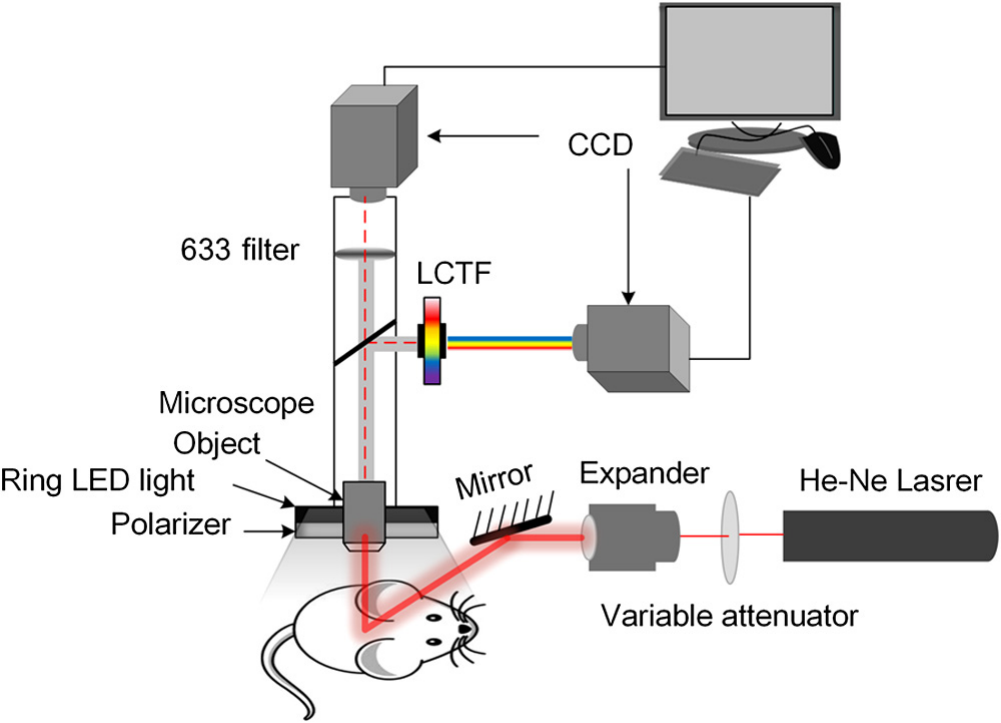
Schematic of the imaging system.

Here, we combined the LSCI and HSI dual-mode imaging system to monitor blood flow and blood oxygen simultaneously. This system mainly consists of two charge-coupled device cameras (Pixelfly USB, PCO Company, Germany), a liquid crystal tunable filter (LCTF, CRi Varispec VIS, PerkinElmer, Waltham, Massachusetts), a stereo microscopy (SZ61TR, Olympus, Japan), a ring-like LED light with a polarizer and a filter (633±5  nm), and a He-Ne laser beam (λ=632.8  nm, 3 mW) expanded by a collimating lens as shown in Fig. 1. The bandwidth of LCTF is 7 nm. A certified reflectance standard (SRS-99-020, Labsphere, North Sutton, New Hampshire) was used to acquire the standard hyperspectral images. All the hyperspectral images were acquired from 500 to 620 nm (with a step size of 10 nm). The skin vascular blood oxygen saturation can be obtained from the hyperspectral dataset using the algorithm developed previously,^40^ and the artery and accompanying vein can be distinguished according to the blood oxygen level. Based on the recorded raw speckle images, the corresponding blood flow velocity maps can be obtained by laser speckle temporal contrast analysis method.^47^^–^^49^

We used LSCI and HSI to obtain intact skin images of the dorsal region of interest before DSOCA treatment. Then, the optical clearing skin window was established for imaging skin microvessels. Under the help of optical clearing skin window, the skin vascular blood flow and blood oxygen images were recorded for 5 min at 1-min interval before injecting NE. Then, we injected 150-μL NE (0.2 mg/mL) via tail vein and monitored the vascular blood flow and blood oxygen for 45 min at 1-min interval. Furthermore, eight mice in each group (non-T1D, T1D-1w, T1D-2w, and T1D-4w) were used as the number of statistical data. To illustrate whether optical clearing skin window affects the blood flow and blood oxygen, we also monitored the skin vascular blood flow and blood oxygen simultaneously before and after injecting 150-μL saline via tail vein for the non-T1D.

### 2.4 Measuring the Expression Level of α1-Adrenergic Receptor

To explore the reasons for changes in NE-induced vascular reactivity tests, we measured the expression level of α1-adrenergic receptor (α1-AR) that was the target of NE. The related protein was extracted from *ex vivo* dorsal skin samples of mice (n=9 for each group), which was used for western blot analysis with a rabbit anti-α1-AR antibody (1:500, Santa Cruz Biotechnology #SC-28982) and mouse anti-GAPDH antibody (1:10,000, Tianjin Sungene Biotech Co., Ltd., #KM9002). In this work, the significant differences among different T1D stages were analyzed using student’s t-test with MATLAB.

## 3 Result

### 3.1 Blood Flow and Blood Oxygen Changes for the Non-T1D with Optical Clearing Skin Window

**Fig. 2:**
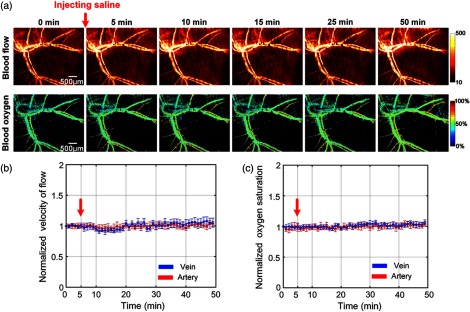
Monitoring vascular blood flow and blood oxygen through optical clearing skin window. (a) Typical skin vascular blood flow and blood oxygen saturation maps before and after injection of saline. The time-lapse changes of (b) the corresponding blood flow and (c) oxygen saturation in artery (red line) and vein (blue line) before and after injection of saline. The red arrows refer to time of injection (n=8, mean ± standard deviation).

To prove whether DSOCA treatment and the experimental operations affect the blood flow and blood oxygen within the reported 50 min of this experiment, we monitored the skin vascular blood flow and the corresponding blood oxygen simultaneously through the optical clearing skin window using LSCI and HSI. Figure 2(a) shows that there is no obvious change in the distribution of blood flow and blood oxygen saturation. Figures 2(b) and 2(c) show that the arteriovenous blood flow and blood oxygen saturation almost kept constant within 50 min of the observation. Thus, it means that DSOCA treatment and the experimental operations for mice hardly changed the vascular blood flow or blood oxygen saturation in both the artery and vein.

### 3.2 Dynamic Response of Blood Flow with the Development of T1D

**Fig. 3:**
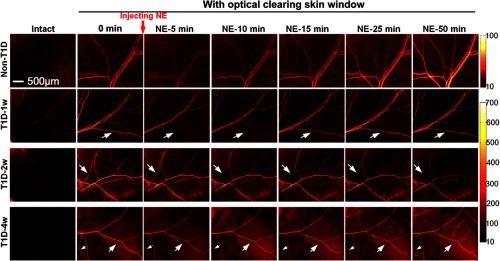
Dynamic monitoring of NE-induced skin vascular blood flow response at different stages of T1D through optical clearing skin window.

We monitored the NE-induced skin vascular blood flow response with the development of T1D. The first column of Fig. 3 shows that the skin vascular blood flow distribution is nearly invisible for intact skin. After establishment of the optical clearing skin window, the vascular distribution can be clearly observed. For non-T1D and T1D-1w, the blood flow evidently decreases at the first 5 or 10 min after injecting NE (as indicated by the white arrows in Fig. 3) and then recovers to initial level. However, for T1D-2w and T1D-4w, NE induces an evident decrease in blood flow, even leads to little blood flow perfusion in some vessels (see white arrows in Fig. 3), and the blood flow does not recover to the initial level after injection of NE (as indicated by the white arrows in Fig. 3).

**Fig. 4:**
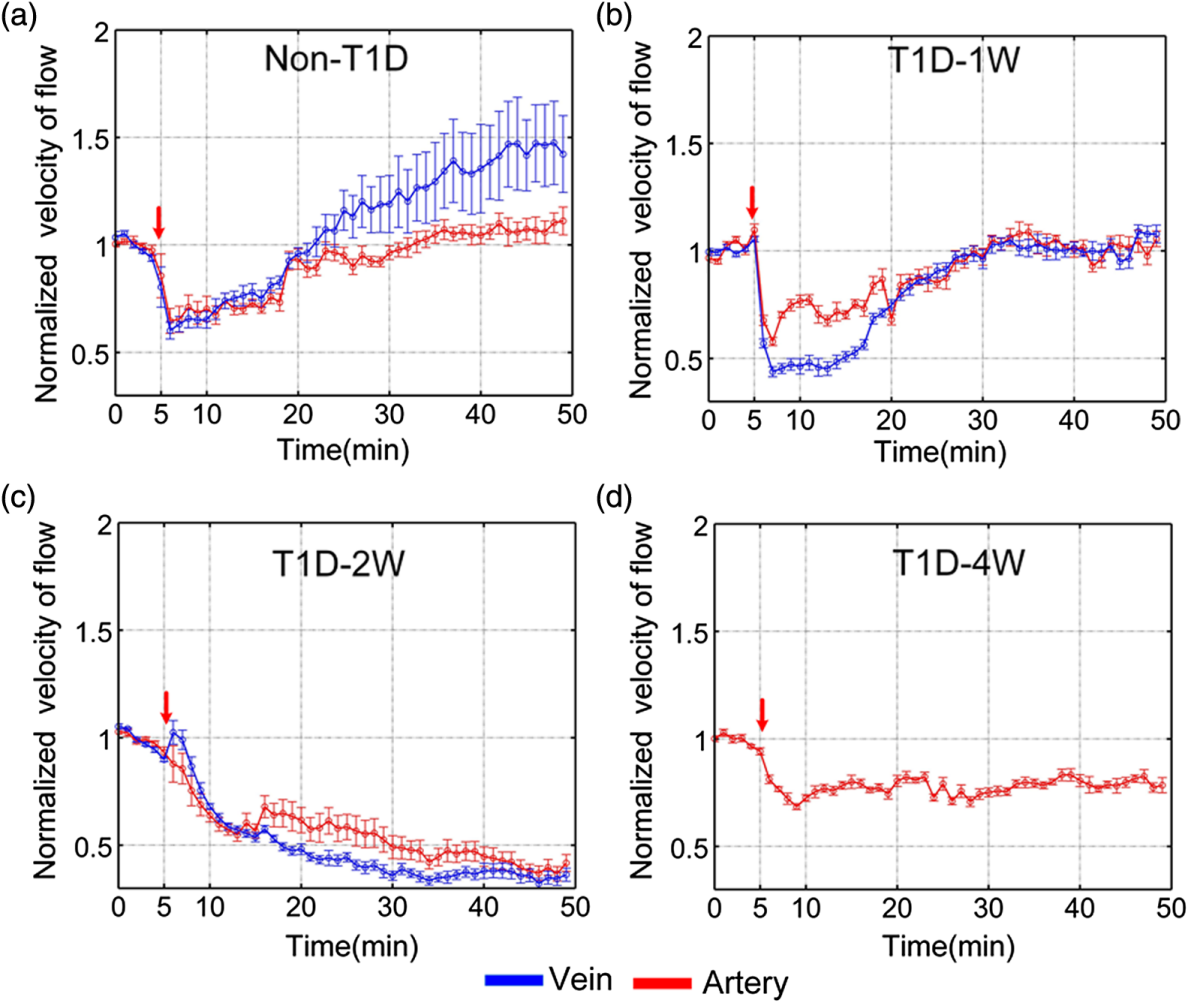
The time-lapse blood flow dynamic responses in artery (red line) and vein (blue line) at different stages of T1D: (a) non-T1D, (b) T1D-1w, (c) T1D-2w, and (d) T1D-4w. The red arrows refer to the time of injection (n=8 for each group, mean ± standard error).

Further, the blood flow responses of arteries and veins to NE are quantitatively analyzed as shown in Fig. 4. For the non-T1D, the blood flow velocity of arteries and veins decreases by 35.2%±5.8% and 40.0%±3.9%, respectively, and almost recovers at about 20 min after injection of NE as shown in Fig. 4(a). However, the venous blood flow velocity of T1D-1w and T1D-2w decreases by 42.1%±1.9% and 56.2%±2.3% as shown in Figs. 4(b) and 4(c), respectively. For T1D-4w, after injection of NE, venous blood flow perfusion almost disappears, which cannot be analyzed effectively. Thus, we only show the result about arterious blood flow velocity in Fig. 4(d). These results indicate that NE induced the decrease of venous blood flow velocity strengthens gradually with the development of T1D. Unlike the venous blood flow changes, the arterious blood flow velocity of T1D-1w, T1D-2w, and T1D-4w decreases by 42.1%±1.9%, 63.3%±3.3%, and 31.5%±1.4%, respectively. For T1D-1w, the blood flow velocity of arteries and veins nearly recovers at about 30 min after injection of NE, but that does not completely recover for T1D-2w and T1D-4w. The results indicate that diabetes will influence the skin blood flow perfusion. With the development of diabetes, its impact will intensify.

### 3.3 Dynamic Response of Blood Oxygen with the Development of T1D

**Fig. 5:**
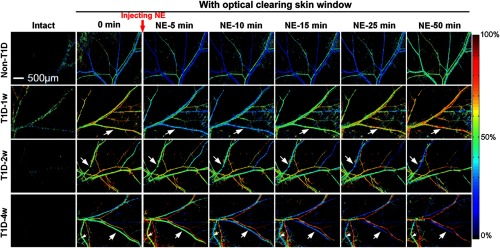
Dynamic monitoring of NE-induced corresponding skin vascular blood oxygen response at different stages of T1D through optical clearing skin window.

The NE-induced corresponding skin vascular blood oxygen responses were also monitored simultaneously by LSCI and HSI. Figure 5 shows that the optical clearing skin window also allows us to obtain much more clear distribution maps of skin vascular blood oxygen, compared to images from intact skin. For the non-T1D and T1D-1w, the blood oxygen decreases after injection of NE at the first several min and then almost recovers. For T1D-2w and T1D-4w, blood oxygen saturation of some veins decreases and keeps at a very low level after injection of NE as indicated by the white arrows in Fig. 5.

**Fig. 6:**
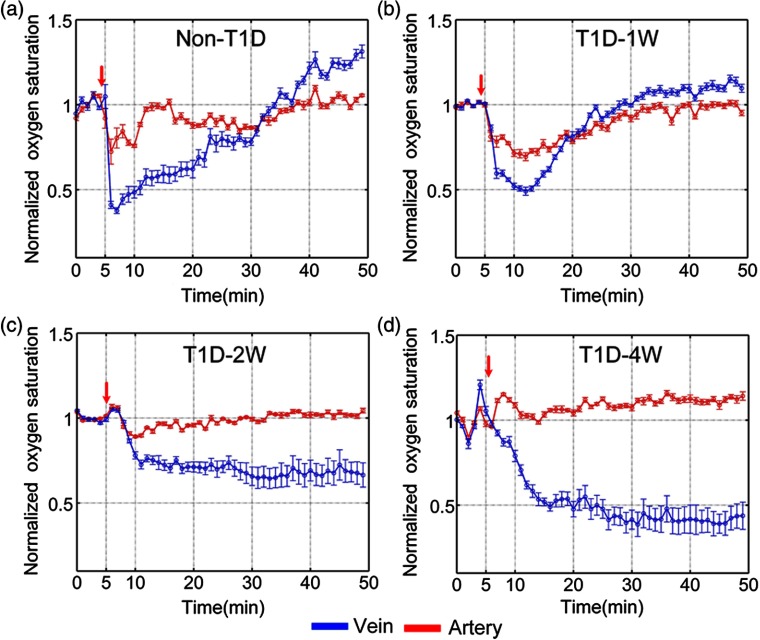
The time-lapse blood oxygen dynamic responses in artery (red line) and vein (blue line) at different stages of T1D: (a) non-T1D, (b) T1D-1w, (c) T1D-2w, and (d) T1D-4w. The red arrows refer to the time of injection (n=8 for each group, mean ± standard error).

In addition, the blood oxygen saturation responses of corresponding arteries and veins were also quantitatively analyzed. After injection of NE, the arterious blood oxygen saturation of non-T1D, T1D-1w, T1D-2w, and T1D-4w decreases by 27.6%±7.3%, 30.6%±2.3%, 10.9%±1%, and 3.9%±0.4%, respectively (see Fig. 6). It means that NE-induced the response of arterious blood oxygen becomes weaker for T1D-2w and T1D-4w than those for non-T1D and T1D-1w. However, for T1D-2w and T1D-4w, the blood oxygen saturation of the vein decreases without recovery after injection of NE. The results indicate that diabetes can also lead to the abnormal blood oxygen response, and there are some differences in the blood oxygen response between arteries and veins.

### 3.4 Expression Level of α1-Adrenergic Receptor

**Fig. 7:**
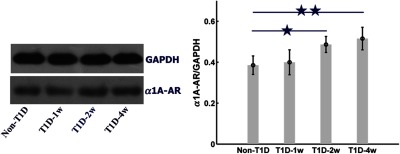
The expression level of α1-AR at different stages of T1D assessed by western blot. ★ p<0.05 and ★★ p<0.01 compared with non-T1D (n=9 for each group, mean ± standard error).

To explore the reason for changes in NE-induced skin vascular functional response, we measured the expression level of α1-AR in skin vascular smooth muscle by western blot analysis. Figure 7 shows that the expression level of α1-AR does not increase significantly for T1D-1w, but it significantly increases for T1D-2w and T1D-4w (p<0.05). The upregulation of α1-AR perhaps leads to increase in vasoconstricting effect to NE, which may cause the blood flow to decrease without recovery for T1D-2w and T1D-4w. It demonstrates that the impaired skin vascular smooth muscle function is related to T1D-induced abnormal expression of α1-AR.

## 4 Discussion

The vasomotor stimulation is a classical vascular reactivity test for evaluating the vascular disorders. Previous studies about diabetes-induced vascular dysfunction focused on *ex vivo* great vessels. Kanie and Kamata^50^ demonstrated that diabetes could significantly enhance the response of the aortic rings to NE. However, the *ex vivo* great vessels might not be consistent with the microvessels in the same reactivity test. Some *in vivo* studies only focused on comparing skin blood vessel function between diabetes and normal physiological state,^15^^,^^16^^,^^51^^–^^54^ but little attention has been paid to changes in vascular dysfunction along the development of diabetes. Krumholz et al.^13^ employed the functional photoacoustic microscopy to measure blood flow and oxygen level of mouse ear blood vessels with the development of diabetes in a resting state. Their results showed that from the second week of T1D, the arterious blood flow velocity significantly reduced but venous blood flow velocity significantly increased at the sixth week of T1D, and there was no significant change in the arteriovenous blood oxygen level. However, in this work, we simultaneously monitored skin arteriovenous blood flow and blood oxygen dynamic responses to NE-induced vasomotor in different stages of diabetes (T1D-1w, T1D-2w, and T1D-4w). Compared with Krumholz et al.’s^13^ results, we found that NE-induced blood flow and blood oxygen responses changed with the development of T1D, and there were some differences in the influence degree of blood flow and blood oxygen response between the artery and vein with the development of T1D.

For the normal physiological state (non-T1D), after injection of NE, the vascular blood flow and blood oxygen can recover to initial level; finally, it was consistent with previous reported results.^55^^,^^56^ It was known that NE could increase the vascular resistance; thus, the blood flow velocity reduced.^57^ With the development of T1D, the NE-induced response progressively increased. This might contribute to a gradual increase in blood flow resistance and even cause occlusion of blood flow eventually. Especially for T1D-4w, the arteriovenous blood flow obviously decreased after injection of NE, which even led to disappearance of the venous blood flow perfusion. It indicated that the sensitivity to NE-mediated vasoconstriction changed due to T1D. With the development of T1D, we found that the expression level of α1-AR (the effect target of NE) upregulated and the adrenergic responsiveness rose, which was consistent with the previous report.^58^ This suggested that diabetes patients should prudently use NE or other vasoconstriction drugs in clinical treatment.

Additionally, NE-induced corresponding blood oxygen response also changed with the development of T1D. However, the arteriovenous blood oxygen response was different for T1D-2w and T1D-4w, and the decrease of arterious blood oxygen induced by NE greatly weakened. After the injection of NE, the blood flow perfusion in some veins almost completely disappeared, and the blood oxygen saturation of corresponding veins was kept at a very low level for T1D-2w and T1D-4w. This is because the poor blood flow perfusion can cause a lack of oxygen delivery, the continuing consumption of oxygen in the veins leads to a very low level of blood oxygen, which means that the change in blood flow and blood oxygen is interconnected. Our results indicated that diabetes could lead to the abnormal blood flow and blood oxygen responses, and there were some differences in the blood flow and oxygen response between arteries and veins. In previous reports, diabetes could cause high glucose concentration and insufficient blood oxygen, which contributed to poor blood circulation.^59^ This might lead to thickening in the basal layers of vascular walls and resulted in poor oxygen delivery. Furthermore, an increase in the clotting ability of blood could lead to some vascular occlusions and limb ischemia.^59^ Therefore, it means that diabetes can impair vascular blood flow perfusion and blood oxygen metabolism with the development of diabetes.

Researcher typically employed various skin imaging windows for observing blood vessels or cells,^23^^,^^24^ but they will inevitably cause a local and even systemic proinflammatory stimulus,^25^ which will affect the vascular structure and function as well as the behavior of immunocytes. Diabetes can accelerate inflammation of wound caused by the surgery, which may affect the vascular function.^42^ Fortunately, *in vivo* skin optical clearing method developed recently,^41^ as a low-cost and convenient method, provides a good way for studying skin microvascular hemodynamics under pathological conditions. Additionally, under the consistent experimental condition, we found that arteriovenous blood flow and blood oxygen saturation almost kept unchanged before and after injection of saline. Thus, it means that skin optical clearing agent or other experimental operations for mice will hardly affect NE-induced changes in vascular blood flow and blood oxygen.

In this work, LSCI was applied to detect NE-induced changes in blood flow perfusion with development of T1D. We found the disappearance of blood perfusion in the vein for T1D-4w due to the microvascular dysfunction of diabetes. Actually, LSCI is widely used to monitor the dynamics of cerebral blood flow, including the disappearance of blood flow perfusion after middle cerebral artery occlusion.^60^ Here, the combination of LSCI and HSI is completely available for monitoring NE-induced cutaneous vascular blood flow and blood oxygen response of mice simultaneously. LSCI, as a low-cost tool, also has been used to assess skin microvascular reactivity in clinic,^19^^,^^61^ even though the spatial resolution suffers from the turbid human skin. In contrast, OCT is a more powerful tool to perform deep-tissue imaging, even for human cutaneous vessels,^62^ which will have ability to study patients with diabetes-induced skin microvascular dysfunction in future.

## 5 Conclusion

In summary, we realized a better visualization of skin microvascular dysfunction in T1D mice by LSCI and HSI with the assistance of *in vivo* skin optical clearing method and investigated NE-induced blood flow and blood oxygen responses in the artery and vein. The blood flow and blood oxygen responses had changed dramatically with development of diabetes. The arteriovenous blood flow decreased without recovery after injection of NE, and the decrease of arterious blood oxygen induced by NE greatly weakened due to the development of T1D. These changes of NE-induced vascular reactivity were related to the upregulation of α1-AR. This study indicated that skin microvascular function can be a potential research biomarker for early warning in the occurrence and development of diabetes. Additionally, *in vivo* skin optical clearing method provides a feasible solution to visualize skin microvascular blood flow and blood oxygen responses under the pathological conditions.
